# Preliminary investigation of acaricidal activity of leaf extract of *Nicotiana tabacum* on dog tick *Rhipicephalus sanguineus*

**DOI:** 10.14202/vetworld.2019.1624-1629

**Published:** 2019-10-25

**Authors:** Taiwo Olaide Oyagbemi, Anofi Ashafa, Johnson Olayide Adejinmi, Oluwafemi Omoniyi Oguntibeju

**Affiliations:** 1 Department of Veterinary Parasitology and Entomology, Faculty of Veterinary Medicine, University of Ibadan, Nigeria; 2 Faculty of Natural and Agricultural Sciences, Qwaqwa Campus, University of the Free State, Phuthaditjhaba, South Africa; 3 Phytomedicine and Phytochemistry Group, Department of Biomedical Sciences, Faculty of Health and Wellness Sciences, Cape Peninsula University of Technology, Bellville 7535, South Africa

**Keywords:** adulticidal, ethnoveterinary, larvicidal, *Nicotiana tabacum*, *Rhipicephalus sanguineus*

## Abstract

**Background and Aim:**

Tick infestation of domestic animals remains a major constraint to livestock productivity across all agro-ecological zones most especially in small animal practice. The most common method of tick control is the use of synthetic acaricide. However, a widespread increase of acaricidal resistance, scarcity and high cost of acaricides especially to farmers of low-income earnings in developing countries support the need for alternative tick control methods. Among the alternative methods for tick control is herbal therapy. In this study, we investigated the acaricidal activity of methanol and N-hexane leaf extracts of *Nicotiana tabacum* against dog ticks – *Rhipicephalus sanguineus*.

**Materials and Methods:**

Larvicidal and adulticidal activity of *N. tabacum* leaf extract were examined on the dog tick – *R. sanguineus* in an *in vitro* experiment using larval packet test and adult immersion test respectively. Phytochemical and Gas Chromatography-Mass Spectrometry (GC–MS) analysis of the leaf extract were also carried out using standard methods.

**Results:**

We observed a tick mortality rate that was concentration-dependent. However, N-hexane extract showed a higher significant acaricidal effect than methanol extract. Lethal dose (LD_50_) of *N. tabacum* was 0.06. High quantity of terpenoids was obtained from *N. tabacum*. Lower tick glutathione S-transferase observed with varying concentration of *N. tabacum*. GC–MS revealed *Pyridine*, 3-(1-*methyl*-2-*pyrrolidinyl*)-, (S) - *Nicotine*, *Citronellyl propionate*, *Crotonaldehyde*, *Lavandulyl acetate*, *trans-Phytol* and *Amitrole* (3-*Amino*-1, 2, 4-*triazole*) in *N. tabacum*.

**Conclusion:**

Both methanol and N-hexane leaf extracts of *N. tabacum* exhibited observable acaricidal property against the larvae and adult *R. sanguineus* of dog.

## Introduction

Chemical control with synthetic acaricides is considered one of the best methods so far, although, ticks have developed resistance against a great number of these chemical acaricides [1]. Controlling of ticks is imperative due to their ability to transmit more pathogenic organisms compared to other arthropod vectors [2]. Great economic loss ranging from low productivity, mortality, and direct effect of tick burden has been attributed to tick infestation [3]. A number of tick resistances to synthetic acaricides have been reported by many researchers [4].

Biodegradability, low toxicity to the environment, and non-targeted species coupled with ready availability of some botanical acaricides give it an advantage over synthetic acaricides [5]. So far, promising results have been obtained from some plants screened for anti-tick properties. Among the natural products, plant extracts and essential oils have shown high significant activity against all the stages of economically important tick species [6]. Plant essential oils have been reported to show ovicidal, larvicidal, pupicidal, adulticidal and repellant activities against *Rhipicephalus* species of tick [7]. Insecticidal and acaricidal activities of neem products have also been reported [8]. *Tephrosia vogelii*, *Ricinus* *communis*, and essential oils of *Syzygium aromaticum* gave a satisfactory result on engorged females of *Rhipicephalus* spp. [9-11]. The involvement of different mixtures of biological compounds in herbal therapy helps to checkmate resistance development [12]. Dipeolu and Ndungu [13] reported an accidental acaricidal effect of leaves of *Nicotiana tabacum* on adult female *Rhipicephalus* spp. *Mangifera indica* [14] and *Azadirachta indica* [15] have also been reported to possess acaricidal activity.

For the purpose of this study, we explored the acaricidal potential of the leaves of *N. tabacum*. It belongs to *Solanaceae* family, which includes crop species such as tomatoes, potatoes, and peppers [16]. Nicotine plant has more than 60 species, among which only *Nicotiana rustica* and *N. tabacum* are widely used by humans. Here, we investigated the acaricidal activity of methanol and N-hexane leaf extracts of *N. tabacum* against dog ticks *Rhipicephalus sanguineus*.

## Materials and Methods

### Ethical approval

This study was approved by the Animal Care and Use Research Ethics Committee, University of Ibadan, Nigeria (UI-ACUREC/App/17/0031).

### Plant material

Plants of *N. tabacum* were randomly collected from different geographical locations of the study area within Ibadan Metropolis from October 2016 to March 2017. The plant was authenticated in the Department of Botany, University of Ibadan, Oyo State, Nigeria, and kept as herbarium specimen with a voucher number UIH-22634.

### Preparation of extracts

The leaves were collected in the dry season, shade dried at room temperature for 2 weeks. The dried leaves were pulverized using a grinder. The powdered leaf material (3 kg) was cold extracted using 10 L each of methanol and N-hexane for 72 h and then concentrated with rotary vacuum evaporator. The extracts which were semi-solid forms were completely dried at room temperature. Different quantity of extracts was weighed and dissolved in seven different dilutions of dimethyl sulfoxide (DMSO) at the rate of 0.04, 0.05, 0.06, 0.07, 0.08, 0.09, and 0.10 mg/ml serial dilution. About 16% diazinon (DZN) (organophosphate) served as a positive control and DMSO as a negative control.

### Collection of ticks

A total of 600 fully engorged adult female ticks *R. sanguineus* were collected from different naturally infested adult dogs brought to University of Ibadan, Veterinary Teaching Hospital with a history of no recent exposure to any acaricide. Maximum of 10 adult female ticks were collected from each infested dog. Ticks were harvested from selected predilection sites with the aid of blunt pointed forceps to avoid any harm to ticks and hosts. Ticks were collected into Bijou Bottles and labeled with details of animals such as sex, breed, age and date of collection. The ticks were reproduced to obtain the larva for larval immersion test. It was not a pooled sample. The collected ticks were singly morphologically identified using taxonomic keys [17].

### Sub-acute toxicity test

Sub-acute toxicity test was conducted according to the Organization for Economic Co-operation and Development guidelines [18]. Group A received a body spray of 0.05 mg/ml DMSO; Group B received 0.05 mg/ml body spray of 16% DZN. Groups C, D and E received body spray of 0.04, 0.06, and 0.08 mg/ml of both methanol and N-hexane leaves extract of *N. tabacum* separately. The rats were exposed once a week for 3 consecutive weeks. The animals were observed daily for any sign of toxicity physically and grossly such as skin reaction, loss of appetite, lacrimation, convulsion, diarrhea and mortality.

### Adult immersion test (AIT)

The effects of adulticidal activity on the ticks were examined in an *in vitro* experiment. Different concentrations of leaf extracts of the plant *N. tabacum* were evaluated on the ticks collected from natural infested dogs. There were seven groups (0.04, 0.05, 0.06, 0.07, 0.08, 0.09, and 0.10 mg/ml serial dilution) containing 10 ticks in each group. The pour-on method was used as described [19]. Organophosphate and DMSO served as positive and negative control group, respectively. The seven groups each of 10 adult female *R. sanguineus* ticks were weighed and dipped in the respective dilutions for 10 min. After immersion, the ticks were placed in separate Petri dishes and kept in a desiccator maintained at 25°C and 80% relative humidity. The mortality of ticks in all groups was recorded after 30 min, 1 h, 2 h, 4 h, and 6 h with respect to the movement of Malpighian tubule and dark coloration of the larva. The test for every dilution was in triplicates.

### Larval packet test

Various dilutions (0.04, 0.05, 0.06, 0.07, 0.08, 0.09, and 0.10 mg/ml) of *N. tabacum* leaf extracts were prepared in a vehicle (DMSO) and the mixture was dispensed on a piece of filter paper separately for each dilution. The filter paper was folded to form a packet and contained 10 (14 days old) larvae of *R. sanguineus* obtainable from hatched eggs of matured female ticks collected. 7–14-day larvae have been reported previously used and at day 14, seed ticks were already developed into larva when observed under microscope. The packets were incubated with CO_2_ under airtight condition at 25°C and 80% relative humidity within exposure time of 24 h. Under natural condition, the larval stage is easily destroyed following adverse environmental condition, within 24 h; more than 80% of the larvae were dead both in nicotine-treated and DZN-treated group. Alive and dead larvae were counted [19]. All the experiments were carried out in three replicates (larval and adult stage of ticks).

### Determination of acaricide resistance using tick glutathione S-transferase (GST) activity

The tick GST activity was determined [20]. 10 µL of tick supernatant and 140 µL of phosphate buffer saline, then, 10 µL of GSH, add 50 µL of 1-chloro, 2,4-dinitrobenzol. The absorption increase at the new wavelength of 340 nm provides a direct measurement of the enzymatic reaction.

### Gas chromatography-mass spectrometry (GC–MS) of the essential oil of N. tabacum

The essential oil of *N. tabacum* was analyzed using GC–MS (Agilent Technologies, Palo Alto, CA, USA) 5973 Network selective detector with column DB23 model number J and W 1222362 with internal diameter of 60 m×250 µm×0.25 µm (250°C Max). 50 µL of the methanol extract of *N. tabacum* was dispensed into 1 ml sample vial and diluted to 1 ml with methanol. The flow rate of 1 ml/min was used with column flow of 0.57 ml/min under 50°C GC temperature. The total runtime was 37 min.

### Statistical analysis

The one-way ANOVA and Turkey’s multiple comparison tests were carried out using GraphPad Prism version 5 for Windows (GraphPad, San Diego, CA). The results were expressed as means ± standard error of mean and the level of significant difference between the control group and the treated groups was determined. The median lethal dose (LD_50_) value was statistically calculated from mortality data [21].

## Results

Toxicity test: There was no sign of toxicity (skin bruise, irritation, salivation, incoordination, ataxia, and mortality) in the treated group.

### Phytochemical analysis

The phytochemical analysis on the methanol leaf extract of *N. tabacum* revealed the presence of saponins, tannins, alkaloids, flavonoids and anthraquinones. A relatively high proportion of terpenoids was obtained from the extract while cardiac glycosides and steroids were absent as shown in Table-1.

**Table 1:** Phytochemical analysis of *Nicotiana tabacum.*

Phytochemical constituents	*Nicotiana tabacum* (Methanol extract)	*Nicotiana tabacum* (N-hexane extract)
Saponins	+	+
Tannis	+	+
Alkaloids	+	++
Cardiac glycosides	_	+
Flavonoids	+	+
Terpenoids	+	++
Anthraquinones	+	+
Steroid	_	+

### Percentage mortality of larvae

**Figure-1:**
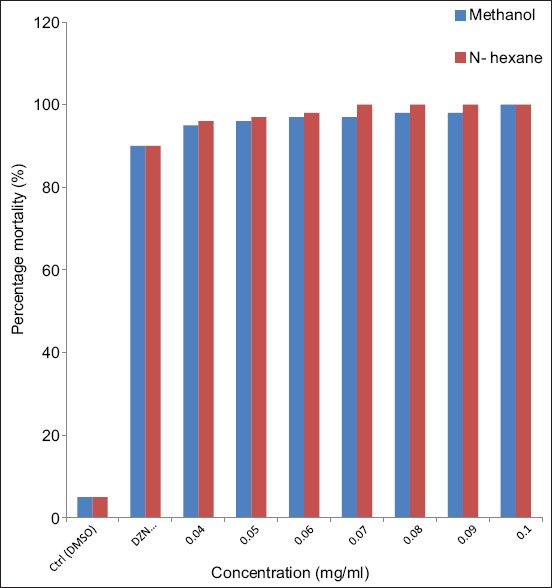
Effect of leaf extract of *Nicotiana tabacum* on percentage mortality of the larval stage of *Rhipicephalus sanguineus*.

The effects of different concentrations of methanol and N-hexane extract of *N. tabacum* on larvae of *R. sanguineus* are shown in Figure-1. Hexane extracts of *N. tabacum* on *R. sanguineus* larvae gave percentage mortality of 99.3% at a dose concentration of 0.07 mg/ml while methanol extracts of the same plant gave the same percentage mortality rate of larvae at a higher concentration of 0.10 mg/ml.

### AIT

**Figure-2:**
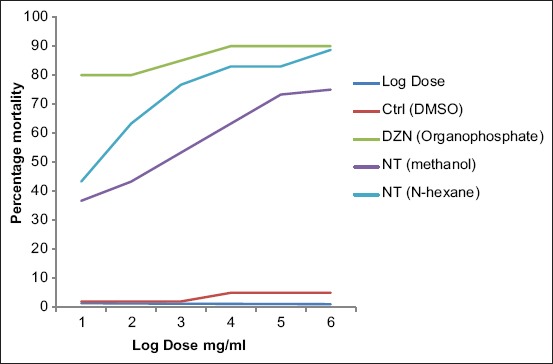
Effect of leaf extract of *Nicotiana tabacum* on percentage mortality of female adult *Rhipicephalus sanguineus*.

The result of the AIT using – N-hexane and methanol leaf extracts of *N. tabacum* is shown in Figure-2. Treatment with organophosphate at 0.05 mg/ml caused 4.5% adult tick mortality and inhibition of fecundity of 79.4%, respectively. The mortality of the engorged adult female ticks, inhibition of fecundity and hatching of eggs were concentration-dependent. The percentage adult female tick mortality varied from 3.67% to 8.87% in methanol extract and 4.33% to 9.33% in N-hexane extract when tested at concentrations ranging from 0.04 to 0.10 mg/ml. The percentage inhibition of fecundity ranged from 25.4% to 92.1% in methanol treated extract and 25.4% to 95.2% in N-hexane treated extracts, respectively. The degree of mortality is directly proportional to increase in concentration.

### Acaricidal resistance

**Figure-3:**
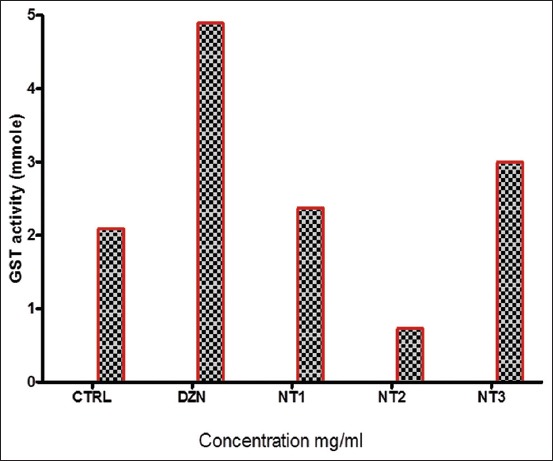
Tick glutathione S-transferase activity of *Nicotiana tabacum*.

GST activity in the tick increased significantly in the DZN-treated group compared to the control and other treatment groups (NT1-NT3) (Figure-3). However, treatment with NT1 and NT2 showed a significant reduction in the activity of tick GST in a dose-dependent manner. The higher the GST activity, the higher the rate of resistance development.

### GC–MS analysis

**Figure-4:**
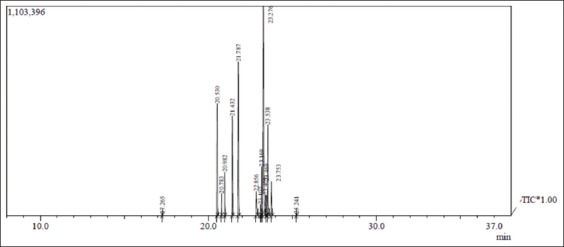
Chromatographic analysis of *Nicotiana tabacum*.

The GC–MS analysis of the essential compound detected from *N. tabacum* includes *Pyridine*, 3-(1-*methyl*-2-*pyrrolidinyl*)-, (S)-*Nicotine*, 2-*Methyl*-4, 5-*dihydrofuran*, *Neophytadiene*, *trans-Phytol*, *Citronellyl* *propionate*, *Lavandulyl acetate*, *Bicyclo* (4.1.0) *heptane*, 3-*methyl*, 2, 3, 4,5-*Tetrahydropyridazine*, *Crotonaldehyde*, 3-*Amino*-1,2,4-*triazole* (*Amitrole*), and 4-*Acetoxy-tetrahydropyran*. Figure-4 showed chromatographic analysis of various bioorganic compounds present in *N. tabacum* with respect to their different retention time and molecular weight.

## Discussion

Phytochemical compounds such as terpenoids and alkaloids were known to possess insecticidal growth-inhibiting, anti-molting, and repellant activities [22]. The potential role of flavonoids in modulating the reproductive functions of ticks has been reported [23]. Alkaloid in the plant extracts was reported to cause mortality and inhibition of fecundity due to its neurotoxic properties [24]. Tannins have been reported to be useful in the treatment of Alzheimer and diabetes due to its hypoglycemic potential [25]. Nicotine is known to act as an agonist for the nicotine acetylcholine (nAch) receptor. Nicotine causes overstimulation of insect’s nervous system resulting in intensive tremors, convulsions and then paralysis [26]. *Pyridine*, 3-(1-*methyl*-2-*pyrrolidinyl*)-, (S)- *Nicotine* is also used as agricultural pesticide [27]. Similarly, *Phytol* is a potent repellant against *Anopheles* *gambiae* [28]. *Citronellol propionate* is a common constituent of some oils reported to have high repellant properties against various insects [29]. *Lavandulyl acetate* and *bicyclogermacrene* were reported as larvicidal agents against three important mosquito vectors [30]. From this study, it is, therefore, reasonable to infer that nicotine affinities for insect’s nAch receptor and additive action of bioactive components have a synergistic effect in tick mortality and inhibition of oviposition. Higher activity of tick GST in DZN-treated group compare to varying concentration of *N. tabacum* is an indication that DZN-treated group is prone to tick resistance which was corrected by *N. tabacum*, especially the NT2 at 0.06 mg/ml [31]. The presence of bioorganic compounds such as *Pyridine*, 3-(1-*methyl*-2-*pyrrolidinyl*)-, (S)- *Nicotine*, *Citronellyl propionate*, *Crotonaldehyde*, *Lavandulyl acetate*, trans-*Phytol* and *Amitrole* (3-*Amino*-1, 2, 4-*triazole*) revealed by GC–MS analysis of N-hexane leaves extract of *N. tabacum* possibly contributed to its potential acaricidal property.

## Conclusion

Based on the results of this study, it can be concluded that leaf extract of *N. tabacum* from different regions within Oyo state, Nigeria, has potential acaricidal activity against the larvae and adult female ticks: *R. sanguineus*. N-hexane leaf extract of *N. tabacum* showed maximum efficacy in various stages of *R. sanguineus*. It is an indication that the non-polar compounds within the N-hexane leaf extract are responsible for acaricidal activity of this plant extract. The mechanism of acaricidal resistance of this novel plant was reported for the 1^st^ time. Possible side effects, especially at the experimental lethal dose of *N. tabacum* against *R. sanguineus*, are under investigation.

## Authors’ Contributions

JOA conceived and designed the plan of research work. TOO carried out the laboratory work and analyzed the results. AA performed the GC–MS analysis; OOO edited and made intellectual contribution to the manuscript. All authors read and approved the final manuscript.
